# A DNA Structural Alphabet Distinguishes Structural Features of DNA Bound to Regulatory Proteins and in the Nucleosome Core Particle

**DOI:** 10.3390/genes8100278

**Published:** 2017-10-18

**Authors:** Bohdan Schneider, Paulína Božíková, Petr Čech, Daniel Svozil, Jiří Černý

**Affiliations:** 1 Institute of Biotechnology of the Czech Academy of Sciences, BIOCEV, Průmyslová 595, CZ-252 50 Vestec, Prague West, Czech Republic; paulina.bozikova@gmail.com (P.B.); jiri.cerny@ibt.cas.cz (J.Č.); 2 Laboratory of Informatics and Chemistry, Faculty of Chemical Technology, University of Chemistry and Technology Prague, Technická 5, CZ-166 28 Prague, Czech Republic; peaters.cech@gmail.com (P.Č.); svozild@vscht.cz (D.S.)

**Keywords:** DNA, DNA-protein recognition, transcription factors, regulatory proteins, histone, nucleosome core particle, molecular structure

## Abstract

We analyzed the structural behavior of DNA complexed with regulatory proteins and the nucleosome core particle (NCP). The three-dimensional structures of almost 25 thousand dinucleotide steps from more than 500 sequentially non-redundant crystal structures were classified by using DNA structural alphabet CANA (Conformational Alphabet of Nucleic Acids) and associations between ten CANA letters and sixteen dinucleotide sequences were investigated. The associations showed features discriminating between specific and non-specific binding of DNA to proteins. Important is the specific role of two DNA structural forms, A-DNA, and BII-DNA, represented by the CANA letters AAA and BB2: AAA structures are avoided in non-specific NCP complexes, where the wrapping of the DNA duplex is explained by the periodic occurrence of BB2 every 10.3 steps. In both regulatory and NCP complexes, the extent of bending of the DNA local helical axis does not influence proportional representation of the CANA alphabet letters, namely the relative incidences of AAA and BB2 remain constant in bent and straight duplexes.

## 1. Introduction

DNA double helix is recognized as the icon of molecular biology for more than 60 years [1]. The ability of DNA to convey the genetic information via self-recognition by base pairing forms paradigm paralleled by its rigor only to physical laws. In contrast to the “digital” mechanism of self-recognition of complementary DNA duplexes, the mutual recognition between DNA and proteins is not driven by a simple code but by a complex combination of structure, electrostatics, and solvation, all of which are ultimately but indirectly determined by the sequences of the interacting molecules. Understanding of protein–DNA recognition is therefore beyond the limits of straightforward complementarity and requires the tools of molecular modeling used to describe analogue protein–protein or protein–small molecule interactions.

Structural features of protein–DNA recognition have attracted a lot of interest [2]. It has been suggested that three-dimensional structures of both interacting biomolecules are equally important and necessary for full understanding of the protein–DNA recognition and that the nucleotide sequence in immediate contact with the protein explains only a few aspects of the recognition process. The importance of the local DNA structure was also highlighted with respect to evolution showing that substantially more DNA regions of the human genome are under selection pressure for maintaining the shape than for the exact nucleotide sequence [3].

A possible approach to comprehend the structural base of biomolecular recognition is to translate complicated three-dimensional structures into a linear code using so called structural alphabets. They simplify an ensemble of possible structures of a suitably selected biomolecular segment into a limited set of building blocks that can be symbolically represented by alphabet letters. The approach is used fairly routinely for describing and analyzing protein structures since it has been suggested [4,5]; a pentapeptide is often used as the biomolecular segment to formulate the alphabet [6]. The approach is however new in analysis of DNA structures. The first DNA structural alphabet has been formulated only recently [7,8]; its first version has been applied to the analysis of protein-DNA interactions [9].

The motivation for this work was to distinguish potentially different structural features of the specifically and non-specifically bound DNA. We examined crystal structures of DNA complexes with regulatory proteins, mostly transcription factors, and DNA in nucleosome core particle (NCP). These two groups of proteins not only exemplify different modes of interaction with DNA but they directly compete for binding to the DNA duplex in the cell. Possible differences in the way how they influence DNA structural behavior therefore bears direct biological consequences: some transcription factors can bind to nucleosomal DNA, while others can only bind nucleosome-free DNA. For instance, the minor groove width is constrained in DNA bound in NCP, precluding thus binding of general transcription factors binding to DNA sequences called TATA box to their wide-open DNA minor grooves [10]. On the other hand, DNA bound in NCP is targeted by a specific group of pioneer factors that recognize and bind the nucleosomal DNA employing mostly the major groove already structurally modified by the histone binding [11]. It has been reported that binding of p53 protein to nucleosomes leads to loss of nucleosome and transcriptional activation in vivo [12]. Direct kinetic competition between DNA binding to nucleosome-forming histones and to transcription factors has also been observed to regulate zebrafish genome activation [13].

The structural behavior of DNA in complexes with regulatory proteins and in NCP was analyzed here by using the Conformational Alphabet of Nucleic Acids, CANA, a first DNA structural alphabet developed earlier [7,8] to catalogue possible dinucleotide structures. Associations between the CANA letters and their dinucleotide sequences displayed different patterns in specifically and non-specifically bound DNA, and thus distinguished these two modes of DNA binding.

## 2. Methods

### 2.1. Selection of Structures

We selected an ensemble of crystal structures that contained 141 protein–DNA complexes of the nucleosome core particle and 942 DNA complexes with proteins classified as regulatory by querying the Nucleic Acid Database (NDB, [14]) release of 2017-03-01 for structures of resolution 3.0 Å or better. The final curated sequentially non-redundant ensemble contains structures with at least one DNA strand longer than six nucleotides and peptide chains longer than 20 amino acids. The analyzed structures are identified by their Protein Data Bank (PDB) codes in the supplementary Table S1. The ensemble consists of 493 structures of DNA in complex with regulatory proteins (hereafter referred to as Regulatory), and 15 structures of the nucleosome core particles (referred to as NCP or Histones). The regulatory proteins in the analyzed ensemble are structurally highly variable and were complexed with DNA of variable but generally limited length. In contrast, the 15 selected complexes of NCP represent a structurally more homogeneous group. NCP is a basic unit of DNA packaging in eukaryotic cells consisting of 146 to 147 base pairs long DNA duplex wound around a histone tetramer of homodimers [15]. The required sequential dissimilarity reduced the number of the analyzed structures from 141 available in the database to 15 that originated from six laboratories to minimize any potential structural bias.

### 2.2. DNA Conformer Classes NtC and the Structural Alphabet CANA

The CANA alphabet letters are assigned to 3′–5′ dinucleotide fragments based on their membership to the conformational classes NtC (for Nucleotide Conformers). The NtC classes are determined by the method of a weighted k nearest neighbors (k-NN) in the 9-dimensional torsion space of seven backbone torsions plus two torsions around the glycosidic bond, which define the base orientation relative to the deoxyribose ring [7,8]. This approach requires a training set of dinucleotides, called a “golden set”, which defines the structures of the 44 NtC classes. The golden set consists of ~4500 dinucleotides that, in a large majority, originate from structures with crystallographic resolution better than 2.0 Å. Determination of NtC classes within the golden set is self-consistent: when a member of the golden set is removed, it is assigned its original NtC class. It is worthwhile to mention that the NtC assignment is only possible for dinucleotides with all atoms, which define the nine analyzed torsion angles, thus excluding incompletely refined DNA segments from the analysis. The NtC and CANA classes were assigned at the web server, dnatco.org, which also contains a full description of all 44 NtC classes and their membership to the complete set of the 12 CANA alphabet letters.

A brief structural annotation of the CANA letters and their numerical presence in the analyzed Regulatory and NCP structures are listed in Table 1. The letters have mnemonic codes: “*A*” indicates that the step exhibits features of the A-form, “B” indicates the B-form; B-A is then a step in which the first nucleotide has B-like features while the second one A-like features. The “canonical”, i.e., the most frequent, A and B forms are labeled as AAA and BBB, miB are structures exhibiting some features typical for B structures, namely C2’-*endo* sugar pucker and high *anti* glycosidic torsion angle, but also untypical structural features, SQX is a letter summarizing non-Z-DNA steps with either base in the *syn* orientation. Because very few dinucleotides adopt conformations characterized by the SQX letter (Table 1), they are not considered for the analysis.

### 2.3. CANA/Sequence Matrices

**Figure 1:**
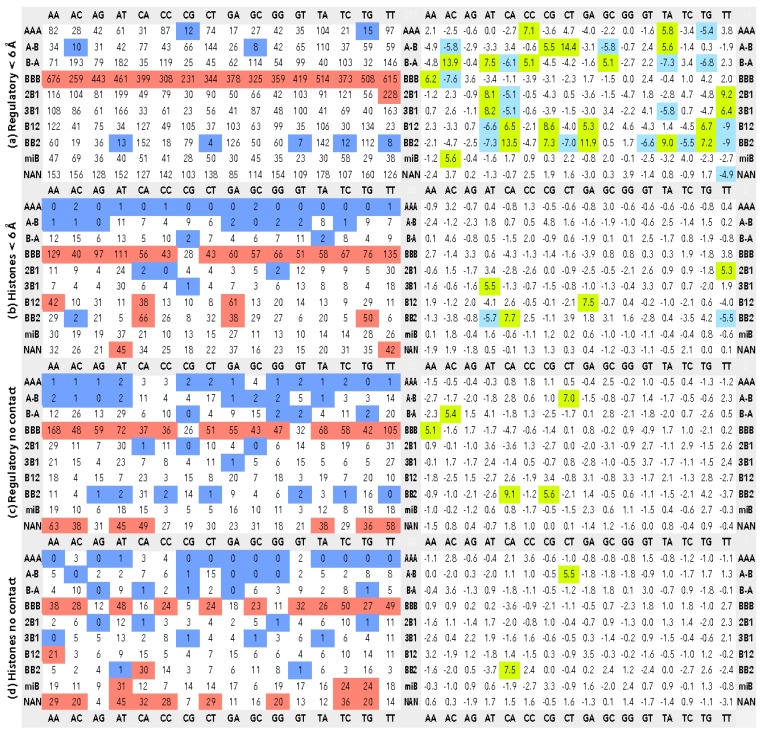
The associations between dinucleotide sequences and structures classified as CANA letters. The matrices in the left column show the instances of the observed CANA/sequence associations, the right column the corresponding standardized Pearson residuals (SPR). Matrices (**a**) and (**c**) show statistics for DNA in complexes with regulatory proteins, (**b**) and (**d**) for DNA in the nucleosome core particles; (**a**) and (**b**) concerns dinucleotides in contact with proteins. Dinucleotides in contact with proteins are closer than 6.0 Å from an amino acid. Matrices (**c**) and (**d**) show data for dinucleotides not in contact with protein. The red highlight shows CANA/sequence associations with twice as many observations as the average for the dinucleotide group; the blue highlights associations smaller than 15% of the average. Green (blue) highlights show SPR with probability less than 1.0 × 10^−6^ for over- (under-) populated associations. Editable version of the figures is in Supplementary Table S3 in the XLSX format so that the color highlights can be modified.

Steps with the assigned CANA letters were further sorted to 16 classes by possible dinucleotide sequences (AA, AC, AG, AT, CA, CC, CG, CT, GA, GC, GG, GT, TA, TC, TG, and TT), and the corresponding counts of the CANA/sequence associations were put to Figure 1a–d.

To screen the potential differences between the structures of steps in contact and not in contact with proteins, we calculated the distances between DNA and protein atoms and dinucleotides in (non-) contact were then discriminated. We used two distance limits to decide whether a dinucleotide is in proximity of an amino acid, 3.6 Å and 6.0 Å. The first value counts nucleotides in direct DNA–protein contact; these are mostly hydrogen bonding, van der Waals, and charge–charge interactions. The second, longer limiting distance, takes into consideration also water-mediated DNA–protein contacts that are numerous and of importance [9,16].

### 2.4. Statistical Treatment of the Data

The primary data are numbers of occurrences (incidences) of the CANA/sequence associations for a particular type of dinucleotide (interacting/non-interacting in Regulatory or Histones groups). To gauge the significance of the numerical patterns in the CANA/sequence matrices, we employed Pearson’s Chi-squared Test as implemented in the R [17] function *chisq.test* from *stats* package. For a given contingency table (contg_table), we obtained Standardized Pearson Residuals (SPR, [18]), which are residuals adjusted to have asymptotic standard normal distribution, from the R function *chisq.test(contg_table)$stdres*. The corresponding probability values were calculated as *chisq.test(contg_table)$p.value*. SPR for each CANA/sequence combination was calculated from 2 × 2 contingency tables.

Pearson residuals are used to evaluate the homogeneity in distribution within a dataset. We aim at distinguishing the homogeneity of distribution of the CANA/sequence associations. Standardization of the residuals allowed us to compare values within the whole data set belonging to many CANA/sequences categories with different numbers of occurrences.

**Figure 2:**
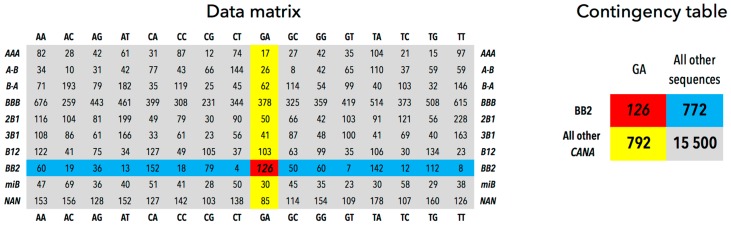
Construction of a contingency table. The BB2/GA association in complexes with regulatory proteins has 126 incidences (red matrix element), the other BB2 letters account for 772 (blue highlight), and the sequences other than GA for 792 (yellow highlight) incidences. There are 15,500 incidences of the remaining CANA/sequence combinations (grey).

The contingency tables were constructed for all CANA/sequence associations from the number of a particular association, the sum of occurrences of the analyzed CANA in the remaining 15 sequences, the sum of occurrences of the analyzed sequence in the remaining nine CANA letters, and finally, the number of observations of the remaining CANA in the remaining sequences; the construction of a contingency table is illustrated in Figure 2 for the BB2/GA association in the dinucleotide group Regulatory < 6 Å.

SPR *r_11_* is calculated from a contingency table using the formula:

(1) $$r_{11}=\frac{obs_{11}-exp_{11}}{\sqrt{exp_{11}*\left(1-p_{c1}\right)*\left(1-p_{1r}\right)}}$$

The variable *obs_11_* in formula (1), is the number of observed occurrences for a particular CANA/sequence association in the element 11 (first line, first column) of the contingency table; the *exp_11_* is the expected number of occurrences in the element 11. It is calculated using the formula:

$$exp_{11}=p_{c1}*\overset{2}{\underset{r=1}{\sum }}obs_{1r}$$

The *p_1r_* and *p_c1_* are the fraction of the first row and column, respectively, from the whole contingency table:

$$p_{c1}=\frac{\sum_{c=1}^{2}obs_{c1}}{\sum_{c=1}^{2}\sum_{r=1}^{2}obs_{cr}}\;\;p_{1r}=\frac{\sum_{r=1}^{2}obs_{1r}}{\sum_{c=1}^{2}\sum_{r=1}^{2}obs_{cr}}$$

The ordinary Pearson residuals *e_cr_*:

$$e_{cr}=\frac{obs_{cr}-exp_{cr}}{\sqrt{exp_{cr}}}$$

are then standardized by their estimated standard deviations based on the expected count in the χ^2^-test:

$$\chi^{2}=\overset{2}{\underset{c=1}{\sum }}\overset{2}{\underset{r=1}{\sum }}\frac{{\left(obs_{cr}-exp_{cr}\right)}^{2}}{exp_{cr}}$$

where the indices *r* and *c* refer to the row and the column of the contingency table.

For example, the contingency table for the BB2/GA association in the dinucleotide group Regulatory < 6 Å is shown in Table 2.

The construction of the table is also shown in Figure 2, data are in Figure 1a. The values of *p_c1_* and *p_1r_* are calculated as:

$$p_{c1}=\frac{126+772}{126+772+792+15500}=0.052\;p_{1r}=\frac{126+792}{126+772+792+15500}=0.053$$

The expected value of the occurrences of the GA sequence in the conformation BB2, the variable *exp_11_*, is calculated as the ratio *p_c1_* times the first column of the table:

$$exp_{11}=0.052\times \left(126+792\right)=47.96$$

Now we have all the variables to calculate SPR for the BB2/GA association:

$$r_{11}=\frac{126-47.96}{\sqrt{47.96\times \left(1-0.052\right)\times \left(1-0.053\right)}}=11.90$$

The SPR value of 11.9 indicates that occurrences of dinucleotides with sequence GA and structure described by the BB2 letter significantly violated the null hypothesis of homogeneity of the matrix elements, the corresponding probability value of the χ^2^ distribution was 3.00 × 10^−32^. When compared to the other CANA/sequence instances, the BII-DNA is “overpopulated” in GA sequences of DNA in contact with regulatory proteins.

A large SPR value indicates an over-representation of the CANA/sequence combination as compared to the null hypothesis; a large negative value would indicate an under-representation. The null hypothesis of the test is that a matrix element representing a particular CANA/sequence combination is as likely as the other combinations. SPR values greater than 3 are usually considered to indicate a lack of fit of the null hypothesis in a particular cell. However, such high values are more likely as the size of analyzed matrix increases from the 2 × 2 dimension of the contingency table. The dimension of our matrices is much larger, 10 × 16, so that matrix elements with SPR values just slightly above the value of 3 or with corresponding χ^2^ distribution probabilities more than 1.0 × 10^−5^ are not considered significant.

In addition to gauging the homogeneity of observations within the analyzed groups of dinucleotides, such as those found in contact with histone proteins (Histone < 6 Å), we wanted to compare distributions of the observations between the dinucleotide groups, for instance between dinucleotides in contact with histone and in contact with regulatory proteins. In this case, we were comparing occurrences in matrices for dinucleotide groups Regulatory < 6 Å and Histone < 6 Å. Contingency tables for these tests can be constructed in two ways. The first compares sequence preferences of individual CANA letters, the second compares the preferences of individual sequences for the CANA letters. These SPR matrices will be referred to as inter-group tables.

Below we show examples of contingency tables comparing dinucleotides in groups Regulatory < 6 Å and Histone < 6 Å for the BB2/GA association (incidences in Figure 1a,b). Table 3 is constructed to measure the significance of the sequence preferences of the CANA BB2, Table 4 measures the CANA preferences for the GA sequence.

The SPR values for these two tests are 1.8 for the BB2/GA contingency table measuring the sequence preferences, and −0.9 for the table measuring the CANA preferences; neither value indicates a significant difference between the tested groups of dinucleotides. Supplementary Table S2 shows calculated SPR values comparing CANA/sequence associations between all four dinucleotide groups.

## 3. Results and Discussion

We discuss the structural preferences of dinucleotides in contact and not in contact with proteins observed in the structures of DNA crystallized with regulatory and histone proteins. As “contact”, we define a distance between nucleotide and amino acid atoms shorter that 6.0 Å. The distance of 6 Å is selected to include dinucleotides, which contact protein via a water bridge, into the group of interacting dinucleotides. Water-mediated contacts are frequent and the involved nucleotides and amino acids have structural [9] and dynamic [16] characteristics similar to those of directly interacting residues. Data for dinucleotides directly interacting with proteins (interatomic distances ≤ 3.6 Å) can be found in Supplementary Table S3. Tables of the CANA/sequence incidences for dinucleotides closer than 3.6 Å from amino acids are not discussed further because interpretation of these tables led to the same conclusions as structurally and statistically more robust data based on the limiting interaction distance of 6 Å shown in Figure 1 and Supplementary Table S3.

### 3.1. CANA and Sequence Distributions

**Figure 3:**
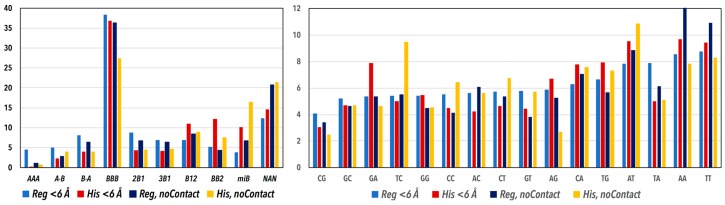
Histograms of the percentages of the CANA letters and 16 dinucleotide sequences in the four discussed groups of dinucleotides: Regulatory < 6 Å, Histones < 6 Å, Regulatory no contact, and Histones no contact. The sequence data are ordered from low to high fractions for the Regulatory < 6 Å group.

Histograms of the percentages of the CANA letters and the dinucleotide sequences in the four dinucleotide groups, Regulatory < 6 Å, Histones < 6 Å, Regulatory no contact, and Histones no contact, are shown in Figure 3. In all four groups, the letter BBB describing the canonical BI conformation numerically dominates. The fraction is however different in the group of “Histones no contact” where it drops significantly from about 37% to 27%. The drop is mostly compensated for by a higher percentage of a mixture of minor and sometimes exotic conformers grouped under the letter miB, and partially by a high occurrence of unassigned conformers NAN. It means that DNA wrapped around histone is deformed from its most likely BI form (letter BBB) more in regions not in contact with protein. Less visible but perhaps more important is observation of extremely low fractions of the AAA letter in all but Regulatory < 6 Å; the A-B and B-A letters are also present more in this structural group. A-DNA form, which is described by the letter AAA, is apparently not compatible with systematic deformation of the histone-bound DNA; it is also rare in DNA not in contact with protein. The last observation concerns relatively high fraction of the BB2 letter, which describes the BII form, in the Histones < 6 Å group. The role of the BII form in histones is discussed below in greater detail.

Sequence frequencies are the lowest for CG followed by GC in all four groups of dinucleotides. The highest proportions are generally observed for the A/T rich sequences, not just in recognition regions of regulatory complexes where it might be expected due to the preference of A and T nucleotides in the consensus regulatory sequences. Dinucleotides of the Regulatory < 6 Å group actually show the least sequential variability; the largest one is observed for the AA step in Regulatory no contact, and for the TC step in Histones no contact. The Watson-Crick pair corresponding to TC, GA, has in contrast low percentage so that TC can be expected to touch NCP proteins less often than GA. A less noticeable but similar situation is observed for the pair CT/AG: CT avoids, while AG prefers contact to NCP. Frequencies of the two-remaining pyrimidine-pyrimidine (Y-Y) and purine-purine (R-R) steps, CC/GG and TT/AA, do not allow generalization that YY steps avoid and RR steps prefer contact with histone. The general trends for sequence preferences of NCP structures has been described previously in greater detail [19,20].

### 3.2. CANA—Sequence Associations

Combining both the structural and sequential information in one matrix (Figure 1) provides a much richer but also more complex picture of the interplay between dinucleotide behavior and the interacting partners. To make the analysis of data in Figure 1 visually more intelligible, we highlighted the low and high instances in color. For the numbers of occurrences (left column), the matrix elements containing less than 15% of the average are marked in blue; those with more than twice as many as the average are marked in red. For the matrices with SPR data, green indicates overpopulation and blue underpopulation with the corresponding probability less than 1.0 × 10^−6^ (probabilities are in supplementary material). Albeit the signal levels used to highlight the data are subjective and arbitrary from the statistical point of view, their variation within fairly large limits does not change the observed patters as can be tested in Supplementary Table S3. The present highlights, therefore, show the characteristic and, we believe, the most important features of the data.

**Figure 4:**
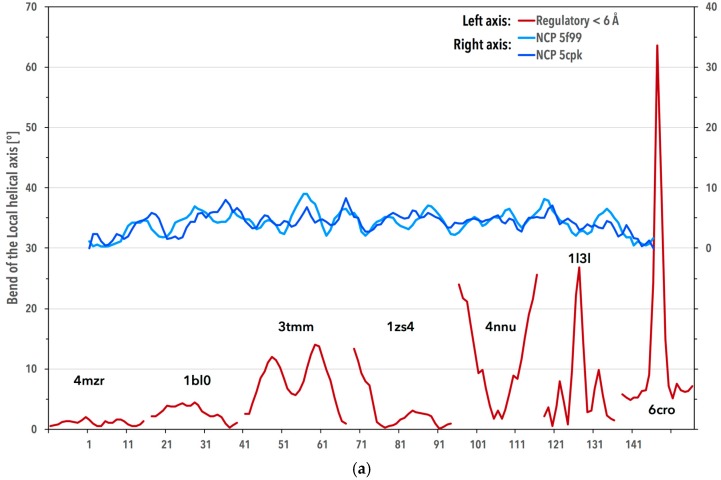

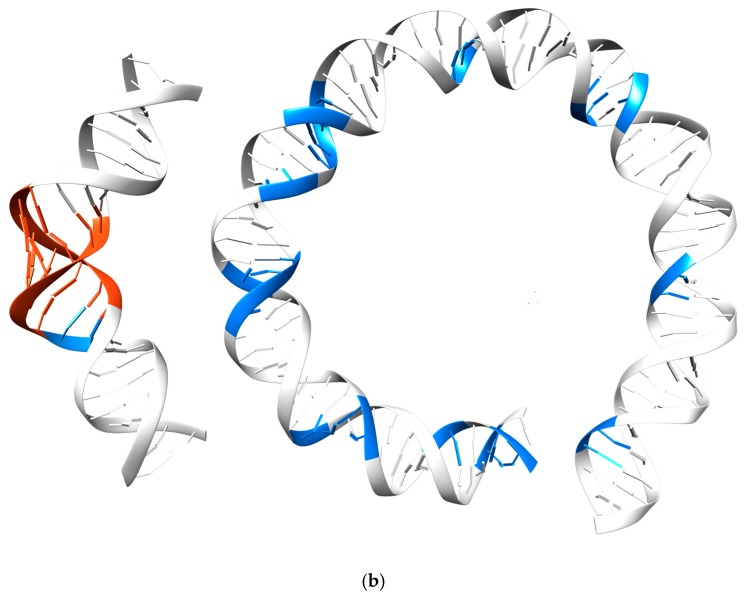
DNA bending in the regulatory complexes and in the nucleosome core particles (NCP). (**a**) Blue lines: the local helical bend of DNA in NCP structures of the PDB codes 5f99 [29] and 5cpk [30]; red lines: the bends for the regulatory complexes 4mzr [31], 1bl0 [32], 3tmm [33], 1zs4 [34], 4nnu [35], 1l3l [36], and 6cro [37]. The local helical axis bend was calculated by the Curves+ program [23] as the *Ax-bend* parameter. The *x* axis denotes the DNA residue numbering in 5f99. (**b**) The left duplex displays DNA from the structure of human TFIIB-related factor 2 and TATA box binding protein bound to U6#2 promoter DNA (PDB code 4roc [21]), where the DNA backbone acquires the A-DNA form (CANA letter AAA) at the bend. The duplex on the right depicts first 75 base pairs from DNA in NCP of the PDB code 5f99 [29]. Dinucleotides adopting the structure described by the CANA letter *AAA* (*BB2*) are highlighted in red (blue) color.

Inspection of the matrices in Figure 1 reveals that except for the numerical prevalence of the canonical BI-DNA form in all four types of dinucleotides, the matrix for the dinucleotides in contact with regulatory proteins, Regulatory < 6 Å, has a different pattern from the other three. It has fairly populated the AAA letter and low numbers of BB2 and miB. The AAA letter is induced at recognition sites of certain transcription factors, where its presence is usually connected to large local deformations resulting in bent DNA duplex. Sharp bending of DNA is known to occur in DNA complexed to TATA box binding proteins, such as in the human TFIIB–TBP–DNA complex (PDB code 4roc [21], Figure 4b). Notable is that the letters AAA, and to a lesser degree A-B, are present in Regulatory < 6 Å in all sequences including A/T rich sequences known not to adopt this DNA form readily.

The Regulatory < 6 Å group differs from the rest also when the standardized Pearson residuals are inspected. It has a check-board pattern of over- and under-represented CANA/sequence matrix elements: e.g., AT and TT are over-represented in 2B1 and 3B1 and under-represented in B12 and BB2, CA behaves inversely. The presence of many significant values in the SPR matrix is caused by a large local variability within CANA rows and sequence columns. It means that interactions in this dinucleotide group, in which contacts are localized to short DNA segments, use a wide spectrum of structures including the A and mixed B/A conformers in a sequence-specific manner.

Structural variability of DNA bound to regulatory proteins is illustrated in Figure 4a by showing the local helical bending of DNA bound to a few transcription factors. While the average bend of the dinucleotide group Regulatory < 6 Å is 2.2°, the actual values fluctuate wildly between 0° and tens of degrees. In contrast, DNA duplex spirals around the nucleosome core in almost two complete turns and obviously needs to be bent repeatedly in small increments sometimes described as “kink and slide states” [22]. The helical axis of 80 DNA steps forming a circle would bend by 4.5° per step on average (360° divided by 80). The actual average of the local helical bend in the 15 NCP analyzed structures, 4.3°, is close to this value with fluctuations between 0° and 9° at individual dinucleotide steps (Figure 4a). The helical axis bend was calculated by the Curves+ program [23] as the per step parameter, Ax-bend.

DNA bending in complexes with regulatory proteins is in most cases realized by structures described by the CANA letters AAA and BB2; specifically, the A-DNA letter AAA is often found at the sites of severe DNA kinks bound to transcription factors (Figure 4b). DNA bending in NCP is not realized by the A-DNA letter AAA, BB2 plays the essential role in its bending. However, the magnitude of the helical bend does not apparently correlate with distribution of the CANA letters (supplementary Table S4). An important class of regulatory proteins, pioneer transcription factors, bind to the major groove of DNA wrapped in NCP [24,25]. DNA complexes of some of these factors, e.g., structures with the PDB codes 1vtn [26], 1puf [27], and 4hje [28], were included into our ensemble of analyzed structures. DNA in contact with protein in these structures shares properties typical for DNA in complexes with most other regulatory proteins, i.e., increased presence of dinucleotides with A-DNA-like features (in these cases the CANA letters A-B and B-A), and most significantly in the BII-DNA form (BB2 letter). Relatively short stretches of DNA in these structures, however, do not allow any deeper analysis of the interplay between the geometry of their major groove and helical bending.

Structures of dinucleotides in contact with histones (Histones < 6 Å group) are characteristic by the frequent occurrence of the letters miB and B12, but most of all by the letter BB2. Sequence preferences for none of these three letters are clear-cut but it fluctuates between high (CA, TG, GA) and low (AC, AT, TC) incidences for BB2. High incidences of the sequences CA and TG and low incidences of GA and TC might point to a preference of pyrimidine-purine over purine-purine and pyrimidine-pyrimidine sequences for BB2. The sequence fluctuation of incidences of BB2 is even higher in the group Regulatory < 6 Å than in Histones < 6 Å DNA, but the fluctuations have a different pattern than in Histones < 6 Å and have no easily explainable sequence pattern. The specific structural role of BB2 in the histone-wrapped DNA is discussed in detail below. The SPR matrix for dinucleotides of the Histones < 6 Å group shows just a few significant values, three of them in BB2. It is important to compare the CANA/sequence distributions of the Regulatory < 6 Å and Histones < 6 Å groups by their inter-group matrices (Supplementary Table S1). They both show significant differences for the BB2 and miB letters pointing again to the essential role of BB2 for the DNA binding.

Both groups of dinucleotides that are not in contact to protein share one feature, high incidence of unassigned conformers NAN without any strong sequence preferences. Many of these dinucleotides are at the strand ends with sufficient freedom to adopt less common structural features, which may sometimes also be induced by the crystal packing forces. Especially in the histone structures, the distribution of the CANA letters does not represent the conformational preferences typical for a free DNA molecule as can be corroborated by a high incidence of conformers with untypical or undefined features, miB and NAN, and low fraction of the canonical BI-DNA, BBB. A question remains whether this is a real structural feature of DNA in NCP, a coincidence of still relatively small sample of available structures, or, as pointed out by a referee of this work, a consequence of poor electron density in the unbound regions of some of the NCP structures.

As the last explanation seems the most likely, we feel that the situation calls for the development of tools to direct the refinement protocols in direction of the optimal agreement with the electron density, but at the same time avoid bias by incorrect or incomplete constraints of the DNA geometry. Such an effort is apparent in the recent development of PHENIX [38] and CCP4 programs REFMAC [39] and EDSTATS [40] that take an advantage of the earlier development of tools to correlate experimental and model electron density such as RSCC [41] and the Uppsala Electron Density Server [42]. The dinucleotide conformer classes (NtC) employed here can help to build more realistic geometric restraints and make the refinement of DNA structures more robust.

A problematic quality of the not-in-contact regions also limits the impact of analysis of protein-DNA binding because it hampers the significance of correlations between the structural behavior of the bound and unbound DNA segments. Further understanding of these correlations would be extremely useful. It could explain the role of exocyclic groups in the major and minor grooves that determine the deformability of the DNA, as it has been observed experimentally [43], as well as extend our insight into the expected facilitation of the protein-DNA binding by sequence-specific deformability of the duplex [44].

### 3.3. Periodicity of the Structural Behavior of DNA in the Nucleosome Core Particle

Our ensemble of the 15 NCP structures contains 32 DNA strands; each of which is about 145 nucleotides long. In all of these DNA strands, longer stretches of the same conformer (the same alphabet letter) are infrequent and dinucleotide steps often alternate between one or two BI and another B-DNA type, most often BII-DNA, or unassigned structure type. In terms of the CANA letters, BBB alternates with BB2, miB, and NAN. These alterations differ slightly from NCP structure to structure and suggest no obvious periodicity.

**Figure 5:**
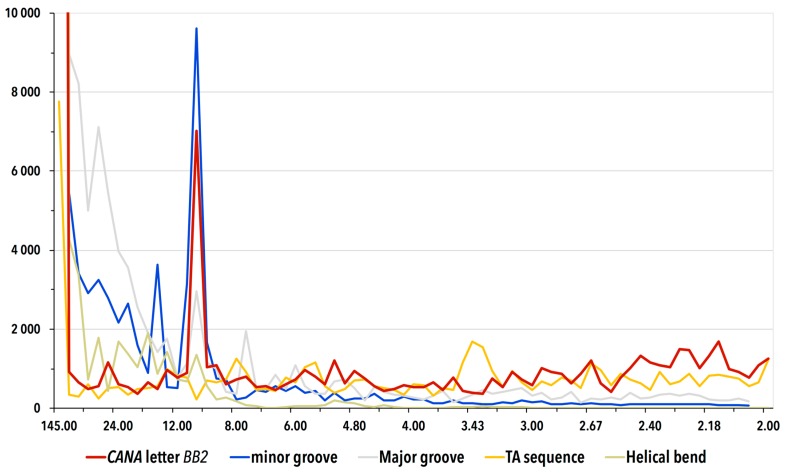
Periodograms of the occurrence of the CANA letter BB2, the minor and major groove widths, TA sequence, and helical axis bend along the DNA in NCP calculated by discrete Fourier transform. The periodograms display the signal averaged from 32 chains in the fifteen analyzed histone structures. The vertical axis measures intensity of the FFT signal, the horizontal axis is labeled as 1/frequency and shows potential periodic behavior. The signal with periodicity of ~10 steps, i.e., 11 nucleotides for the BB2 CANA letter and the minor groove width is strong with intensities well above the values of their respective estimated standard deviations. The high intensity peaks near periodicity of 145 nucleotides are numerical artifacts of the Fourier transform caused by the length of DNA strands, which are about 145 nucleotides long.

In an attempt to reveal the possible periodicity of the duplex bending wrapped around the histone proteins, we Fourier-transformed the presence of CANA letters, dinucleotide sequences, minor and major grove width, and helical bend as a function of the position along the strands (Figure 5). We employed discrete Fourier transformations as implemented in the fft function of Scilab package.

Firstly, we Fourier-transformed each of the ten CANA letters against the remaining nine: the analyzed CANA letter was assigned the value of 1, the other letters the value of 0 and so called periodograms were calculated for all 32 DNA strands. In each NCP strand, we observed a strong signal with a periodicity of about 10 steps for the letter BB2 and the signal became prominent after averaging all 32 periodograms. No other CANA letter provided a signal of significant intensity. The exact periodicity of the *BB2* signal slightly depends on details of the analysis, the average value is 10.3 steps. Because a B-DNA duplex makes one full turn each ~11 nucleotides (~10 steps), the discovered periodicity in the structure characterized by the CANA letter BB2 occurring every duplex turn explains how the DNA wrapping is carried out by the backbone atoms.

Further, we investigated whether any of the 16 dinucleotide sequences provides a periodic signal but we obtained no significant response. The situation is surprising especially for the TA sequence because several previously published studies have indicated a certain ability of the TA sequence to potentiate DNA binding to NCP. A clear and strong sequence signal has been observed by Lowary and Widom for the TA sequence [45]. In their thorough study, they have subjected DNA oligonucleotides to SELEX directed evolution to identify sequences binding with the highest affinity to the histone core [45]. Their analysis, based on Fourier-transforming the resulting sequences, has convincingly demonstrated that the optimal binding between the histone proteins and DNA is achieved for DNA with TA steps dispersed regularly every 10 to 11 steps. An important independent confirmation of the preference for the TA periodicity has been shown in a recent genome-wide study [46]. The sequence periodicity is accepted as an important factor of nucleosome positioning despite its weak pronouncement; several positioning patterns facilitating the bends were suggested including the 10–11 base pair periodicities of AA–TT–TA/GC dinucleotides [47,48] or R5Y5 positioning motif [48,49].

The lack of evidence supporting the periodic presence of the TA sequence in NCP structures is even more puzzling because the BB2 letter, which does behave periodically, is overpopulated in the TA sequence, but in Regulatory < 6 Å, not in NCP structures (Figure 1). The periodic placement of TA or any other sequence seems therefore not the condition but a preference strengthening the binding of DNA in NCP.

Fourier-transformation of the minor groove widths provides a strong signal with the same periodicity as BB2 (Figure 5). The groove width is, however, a consequence of the bending, not its structural carrier as the periodicity of the backbone conformational behavior described above. Also, the previously reported periodic alteration of twist, roll, and tilt [50] is a consequence but not the cause of the bending: “this is only an indirect description that does not address the underlying localized constraints on double helix structure, which moreover arise from a form of protein association that is unique to the nucleosome” [51]. Neither the width of the major groove nor the local helical bend provided periodic signal despite that especially the values of the helical bend oscillate. The values oscillate but the oscillations are apparently not periodic.

## 4. Conclusions

Representation of the DNA structure by the CANA structural alphabet [8] demonstrated its usefulness by revealing significant structural differences between DNA in complexes with regulatory proteins and in the NCP. Different patterns of associations between 16 dinucleotide sequences and their assigned CANA letters can be interpreted as features discriminating the specific and non-specific binding of DNA to proteins. Especially noteworthy is the role of two DNA structural forms, A-DNA and BII-DNA, which are represented by the CANA letters AAA and BB2. The AAA structures are avoided in non-specific complexes with NCP, where BB2 plays the essential role. The wrapping of the duplex around the histone proteins can be explained by the periodic occurrence of the CANA letter BB2 every 10.3 steps along the DNA strand.

In contrast, a role of specific dinucleotide sequences in helix wrapping around NCP could not be confirmed by the available structural data. DNA in complexes with regulatory proteins acquires quite often the A-DNA form. The AAA letter was observed also for some A/T rich sequences (TA, AA, TT) in contact with proteins. In both regulatory and NCP complexes, the extent of bending of the local helical axis does not influence the proportions of the CANA alphabet letters in a measurable way, namely the proportion of the AAA and BB2 letters remains constant in the bent and straight duplexes.

A high incidence of unassigned or untypical conformers (e.g., the letter miB) and lower occurrence of the most typical DNA structure type, BI-DNA, in DNA regions not bound to proteins indicates limits of the available refinement tools and the need of their integration with the validation tools to direct the refinement process by the combined use of the best geometrical restraints and correlations with the electron density maps.

We showed that plasticity of the DNA double helix can be described by the DNA structural alphabet, and characterized different binding strategies of DNA sequences specifically recognized by regulatory proteins and bound nonspecifically in the nucleosome core particle.

## Figures and Tables

**Table 1:** Numbers of letters of the dinucleotide DNA alphabet CANA observed in the analyzed complexes of regulatory proteins and in nucleosome core particle (NCP). The incidences of the alphabet letter SQX were not analyzed, no Z-DNA letter ZZZ was observed.

The Main Structural Features of the CANA Letters	CANA Letter	Regulatory		NCP	
		#	%	#	%
A-form conformers	AAA	802	4	20	0.4
conformers bridging A- to B-form	A-B	925	4.7	133	2.9
conformers bridging B- to A-form	B-A	1564	7.9	184	4
the most frequent “canonical” B-form	BBB	7559	38.1	1548	33.7
less populated BI conformer	2B1	1692	8.5	204	4.4
less populated BI conformers with switched values of torsions α and γ	3B1	1346	6.8	201	4.4
conformer bridging BI- to BII-form	B12	1380	6.9	475	10.3
BII conformers	BB2	1005	5.1	490	10.7
various minor B conformers	miB	827	4.2	565	12.3
conformers with bases in *syn* orientation, may occur in quadruplexes, other non-duplexes	SQX	69	0.3	0	0
non-Assigned Steps	NAN	2688	13.5	778	16.9
All Steps	ASt	19,857	100	4598	100

**Table 2:** The contingency table for the BB2/GA association in the dinucleotide group Regulatory < 6 Å.

	GA	All Other Sequences
BB2	126	772
All other CANA	792	15,500

**Table 3:** An example of contingency table comparing dinucleotides in groups Regulatory < 6 Å and Histone < 6 Å for the BB2/GA association (incidences in Figure 1a,b). The table is constructed to measure the significance of the sequence preferences of the CANA BB2.

	GA	All Other Sequences
Regulatory < 6 Å BB2	126	772
Histone < 6 Å BB2	38	332

**Table 4:** An example of contingency table comparing dinucleotides in groups Regulatory < 6 Å and Histone < 6 Å for the BB2/GA association (incidences in Figure 1a,b). The table is constructed to measure the significance of the CANA preferences for the GA sequence.

	BB2	All Other CANA
Regulatory < 6 Å GA	126	792
Histone < 6 Å GA	38	201

## Supplementary Materials

The following are available online at www.mdpi.com/2073-4425/8/10/278/s1, Table S1: Analyzed crystal structures identified by their respective PDB codes: 493 DNA complexes with regulatory proteins and 15 DNA in the nucleosome core particle (NCP), Table S2: Standardized Pearson residuals (SPR) gauging differences between the four analyzed groups of dinucleotides. Two sets of SPR values were calculated, for contingency tables sorted by the CANA letters (left) and by the sequences (right). The matrix elements can be highlighted by probability values; Table S3: The associations between dinucleotide sequences and structures classified as CANA letters as in Figure 1 in the main text. The matrix elements in the right column can be highlighted by multiples of the average for the associations, in the right column by probability values as in Table S2. Data are shown for the four analyzed groups of dinucleotides, Regulatory < 6 Å, Histones < 6 Å, Regulatory no contact, Histones no contact, plus for dinucleotides closer than 3.6 Å from atoms of amino acid residues of the analyzed regulatory proteins, Table S4: Relationship between the helix bending and distribution of the CANA letters. Shown are incidences and percentages of the CANA letters in dinucleotide groups Regulatory < 6 Å and Histone < 6 Å. Shown are data for all dinucleotides and for those dinucleotides that are in DNA segments bent by the specified angle. The local helical axis bend was calculated by the Curves+ program as the Ax-bend parameter.
